# Correction: Similar Health Benefits of Endurance and High-Intensity Interval Training in Obese Children

**DOI:** 10.1371/journal.pone.0105486

**Published:** 2014-08-06

**Authors:** The PLOS ONE Editors

It has come to our attention that the Academic Editor who handled this article, Dr Reury Bacurau, is affiliated at the University of Sao Paulo, the same institution where the authors are based. In line with the PLOS ONE competing interests policy (http://www.plosone.org/static/competing.action) we consider this as a potential competing interest. Dr Bacurau was invited by PLOS ONE to act as Academic Editor for this manuscript and we acknowledge that the journal should not have approached him to handle this manuscript.

In the light of this potential competing interest, the PLOS ONE editors have asked an independent member of the editorial board, Yang-Ching Chen, to carefully evaluate the peer review process of this article. Dr Chen has confirmed that the final decision to publish was acceptable but considered that additional information should be supplied in relation to methodological aspects of the study, in line with the journal requirements for reporting of clinical trials.

PLOS ONE requires that manuscripts describing clinical trials report details of the trial registration and copies of the study protocol and a completed CONSORT checklist. The study protocol and CONSORT checklist are included in this Correction. The trial has been registered at clinicaltrials.gov with number NCT02143453, the trial was registered retrospectively and the authors confirm that all related ongoing and planned trials have been registered.

The authors have fully collaborated to supply all the information requested by Dr Chen and are reporting additional methodological information as part of this Correction:

1. The inclusion criteria for the study (such as presence of chronic comorbidities) were checked upon medical assessment at the Obesity Clinics (Endocrinology Department, School of Medicine, University of Sao Paulo, Brazil).

2. The randomization was performed using a computer-generated randomization code (1:1) with a block of 4.

3. The sample size for the study was in line with others used in previous investigations on the effects of exercise in pediatric populations (1). Although there is a lack of studies involving obese children undergoing high-intensity interval training, the authors performed a sample size estimation using previous data on the effects of endurance training on relative VO2max (one of the main dependent variables in the current study) in a sample of lean children with similar age to that in this sample (2). It was determined that 14 subjects would be needed to provide a power (1 - â error) of 0.95, and assuming an á error of 0.05 and an estimated effect size of 0.56 (2). The calculation was based on the assumption of an F-test for repeated measures (within- and between-group interactions). The power analysis was performed using software G-Power - Version 3.1.2.

4. In order to examine the effect of the experimental interventions, the authors employed the "as-received” non-Intent to treat (ITT) approach over the "as-assigned” ITT. The primary research goal of the study was to determine the potential benefits of overlooked interventions (in particular, the high-intensity interval training), and not to determine the value of an existing treatment for the general population. In addition, the estimate of the intervention effect may be diluted using ITT analyses due to the non-compliant, dropouts and compliant participants being mixed together. Moreover, heterogeneity may be presented if non-compliant, dropouts and compliant participants are mixed together in the final analysis. Concerns have been raised that traditional ITT analysis may be too cautious and more susceptible to type II error (3; 4).

## Supporting Information

File S1 Study Protocol - Araujo et al. (2012)
(PDF)

File S2 CONSORT checklist - Araujo et al. (2012)
(PDF)
