# Supplementary material for: Correction: Similar Health Benefits of Endurance and High-Intensity Interval Training in Obese Children
Source: PLoS One. 2014 Aug 6;9(8):e105486. doi: 10.1371/journal.pone.0105486 (PMC4123966; doi:10.1371/journal.pone.0105486)
Supplement: File S1 — Study Protocol - Araujo et al. (2012) (PDF) [file pone.0105486.s001.pdf]

Ana Carolina Corte de Araújo (pesquisadora)

Bruno Gualano (orientador)

Ana Lúcia de Sá Pinto (co-orientadora)

Study of metabolic, anthropometric and cardiopulmonary responses in obese children undergoing interval training compared to endurance training.

São Paulo

2010

1 Introduction

1.1 Obesity

The prevalence of childhood obesity has substantially increased in the last decades , and is currently featured as a worldwide epidemic (STYNE , 2001) . In the early 1990s, the World Health Organization (WHO) estimated the prevalence of eighteen million children under five years overweight (CHILDHOOD , 2001) . A U.S. study showed that , in the years 2003 and 2004 , the frequency of obese children in the U.S. was 17 % (OGDEN , 2005). In Brazil , according to (IBGE , 2006) , the percentage of children overweight was 16.7 % in 2002-2003 , and is more frequent in boys (17.9%) than in girls (5.4%).

The Body Mass Index (BMI), weight [kg] / height [m²], is the parameter of choice to identify obesity in adults and has been validated for use in children and adolescents. Childhood obesity is considered when BMI exceeds the 95th percentile in children of the same age and sex, while overweight is characterized by a BMI between the 85th and 95th (BARLOW , 2007) .

Obesity is associated with several comorbidities in children and type 2 diabetes mellitus (DM II) , menstrual irregularities , muscle -skeletal disorders , sleep apnea , problems such as depression, low self -esteem and metabolic syndrome (COMMITTEE, 2003; DANIELS 2005).

In 2001 , the National Cholesterol Education Program (NCEP) Adult Treatment Panel III (EXECUTIVE , 2001) together with the World Health Organization , established five parameters for the diagnosis of metabolic syndrome in adults : waist circumference (> 102 cm in men and > 88cm in women); triglycerides (≥150mg/dL) ; HDL cholesterol (< 40mg/dL in men and < 50mg/dL in women); blood pressure (≥ 130 mmHg or ≥ 85 mmHg) and fasting glucose (≥10mg/dL), now modified to ≥ 100mg/dL as recommended by the American Diabetes Association (2004) .

In children , the metabolic syndrome is not well defined due to lack of studies and establishing consensus diagnostic criteria (Weiss , 2004) .

The impact of obesity on the musculoskeletal system is still poorly studied . Changes as epiphysiolysis femoral (SCFE) and Blount head disease, which occurs due to an abnormal growth of the proximal tibia resulting in varus deformity have been described (Chan , 2009) . In addition , obesity has a negative impact on the osteoarticular health promoting biomechanical changes in the lumbar spine and legs (PINTO, 2006).

1.2 Exercise

The practice of regular exercise promotes health benefits of obese children as well as in adults . The recommendation is thirty to sixty minutes of physical exercise per day at a moderate intensity. Moreover , it is advisable to restrict sedentary leisure activities for two hours daily (DANIELS , 2005) .
Intermittent training consists of a series of repeated sessions of exercise alternated with periods of recovery. The range of work is part of the training represented by an intermittent high-intensity exercise . The recovery period may include complete rest or light to moderate exercise (FOSS, 2000) .
Intermittent training is done quite frequently in the preparation of athletes because it allows the coach to individualize a specific training program , specifying the predominant energy system in the sport and also induces less fatigue . In addition, intermittent training programs can be modified based on schemas travel to locations and facilities since they need less time to achieve the proposed objectives (Gibala , 2008).
In adult patients with chronic, intermittent training emerges as a promise for better results from metabolic parameters. In 2008 , a study showed that intermittent training in adults with metabolic syndrome resulted in a significant improvement in aerobic capacity and metabolic parameters in these subjects when compared to a control group undergoing continuous exercise. Another study with chronic cardiac patients also showed an excellent result to intermittent training (TJØNNA, 2008).
However, one does not know if the intermittent exercise by requiring high intensity, may present some risks to the patient . Risks such as articular by

weight overload or cardiac arrhythmias (Wisløff , 2007) lesions.
In children , intermittent exercise is little studied , but one study found a greater acceptance of this method , being more dynamic and therefore more playful (Baquet , 2002) .
We observe , therefore, the need for studies attempting to define the metabolic response to intermittent exercise in certain groups such as children, and even the safety of this type of training .

2 Objectives

2.1 Primary Objectives

a) To study the metabolic , anthropometric and cardiopulmonary changes in obese children compared to intermittent training them with the response to ongoing training ;
b) To assess the safety of intermittent training in obese children .

2.2 Secondary Objectives

a) To compare the lipid profile after intermittent training and ongoing training ;
b) To verify glycemic alterations and insulin sensitivity after a period of continuous exercise;
c) To study the behavior of blood pressure and cardiopulmonary capacity gain of obese children after training ;
d) To evaluate the change in aerobic capacity after intermittent and continuous exercise sessions ;
e) To evaluate the effectiveness of intermittent training on continuous through metabolic and cardiopulmonary parameters ;

f) To identify the best acceptance of the child as to what kind of physical training through the grip.

3 Methods

3.1 Study Protocol

Children and adolescents evaluated in this study were followed at League of Childhood Obesity in the Department of Endocrinology , Hospital das Clinicas, Faculty of Medicine, University of São Paulo . This is an experimental prospective study .

3.2 Study Population

Children and adolescent males and females will be studied , including eight to twelve years , with obesity defined by BMI percentile greater than 95 for age and sex , attending the ambulatory League Childhood Obesity , Hospital das Clinicas, Faculty of Medicine, University of São Paulo (US) .
Children will be randomly randomized in 2 groups . The first group will be supervised physical activity performed by the method of continuous training and second , supervised physical activity by the method of intermittent training.
The guardians of the children and adolescents of both groups will receive guidance about the study and will be asked to sign a consent provisions under the rules of the Ethics Committee of the Hospital das Clinicas, University of São Paulo , to enable them to participate in the study .

3.3 Outpatient Program of the League of Childhood Obesity

The program consists of 20 weeks of monthly attendance at the Clinic. Patients receive care from a multidisciplinary team of health : doctors, nutritionists, psychologists and physical education teachers, aiming to promote nutritional education and encouraging physical activity. During follow-up are taught nutrition classes and feeding behavior, as well as consultations with psychologists and conversations with teachers of physical education.

3.4 Anthropometric and body composition

3.4.1 Anthropometric Parameters

Weight: Body weight (kg) measured using a digital Filizola ® , with a maximum capacity of 150 kg with graduations of 100 in 100 grams , with the participants of the survey barefoot and wearing light clothing.
Height : The height (m) measured by a stadiometer calibrated in centimeters and vertical and fixed wooden bar , with mobile bracket for placement on the head of the individual, the same being barefoot , with feet together and to position upright , looking forward.
Body Mass Index : The assessed obesity " Quetelet Index " or Body Mass Index (BMI) , which is defined as the individual's weight (kg) divided by their height (m) squared.
BMI Z score : Calculation that allows quantifying the degree of obesity using the formula : Z score = log 1 / S and (Q / M) , where S is the coefficient of variation for individual age and sex ; Q is the BMI ; M is the median .
Abdominal circumference (AC) : Waist circumference will be determined using non- elastic plastic tape with 0.7 cm wide, the height of the iliac crest .

3.4.2 Method of bioimpedance

The bioimpedance analysis uses the electrical properties of the body to assess body composition. Basically consists of administering an electric current between two points of measuring individual and the opposition to current flow . This opposition depends on the composition of the tissues through which the current (adipose tissue is more resistant to electrical current) . Quantification of bioimpedance allows through known formulas to evaluate the percentage of body water, fat-free mass (lean body mass) and fat mass . The BIA has two components : resistance (R) , or primary opposition to the current flow , which is the opposite of conductance ; and reactance (the opposite of capacitance) which is the ability of altenador store for a short time an electric current (XC2) . The impedance (Z) is represented by the following formula: $Z = \sqrt{(R2 + XC2)}$. To perform the bioimpedance , individuals are adequately hydrated and placed supine on a nonconductive surface , with separate arms torso and legs apart from each other at a distance of 20 cm between the ankles . After cleaning the skin , are placed four electrodes surface (two transmitters and two sensors) . These last two will be placed on the right wrist (at the level of the radial head) and right ankle (the level of the internal malleolus) . The electrodes emit current that are placed far from the previous by about 5 cm . The current that is administered is harmless and is not felt by the individual. After placement of the electrodes , cables, sensors are connected to the monitor and it ends at the electrodes ; are then entered the data on gender , age , height and weight and thus obtained will be a report on body composition , detailing the percentage of fat mass and lean mass . The bioelectrical impedance device used for the assessment of body composition is the Body Composition Analyser (BiaQuantum RJL Systems, Inc , MI , USA) .

3.4.3 Laboratory methods

The peripheral venous blood will be obtained after 12 hours of fasting determination of all laboratory measurements described below .
Glycemia : the determination of plasma glucose will be performed in the

Laboratory. The plasma glucose concentration will be determined in an automatic analyzer (Roche Cobas Integra model), by enzymatic colorimetric method using hexokinase . The reference range considered normal for the glucose is 70 to 100 mg/dL.

Dosage of insulin: plasma insulin concentration will be determined by radioimmunoassay, using the HI - 14K Linco Research , Inc. (USA) kit. Values are expressed as mU/ mL . It will be considered normal values between 5-15 mU / mL . The lowest level of insulin which can be detected by this method is 2mU/mL .

Evaluation of plasma lipids: the total cholesterol (TC), HDL - C and triglycerides (TG) plasma will be performed in automated COBAS MIRA using commercial enzymatic kits from Roche (Mannheim , Germany). The concentration of cholesterol in the LDL and VLDL fractions is determined by the Friedewald where C = VLDL- TG / 5 and LDL-C = TC equation - (VLDL -C + HDL -C).

Evaluation of plasma leptin: leptin plasma levels will be measured by ELISA (Kit EZHL - 80SK , brand Linco Research , Inc., St. Charles , Missouri - USA) .

3.4.4 Evaluation of metabolic parameters

Insulin resistance (fasting insulin): hyperinsulinemia determined by the levels of fasting insulin (Matthews et al , 1985).Pre-puberty individuals will be considered hyperinsulinemic when insulin levels are ≥ 15 mu/ L (TEN; MacLaren 2004), whereas for the puberty individuals the cut-off is 20 Mu/L, since in puberty there is an increase of basal insulin levels (Goran ; GOWER , 2001).

Insulin resistance (HOMA -IR): The Homeostasis Model Assessment (HOMA) is a mathematical model based on the interaction of glucose with insulin. HOMA has been proposed as a method to evaluate the sensitivity or function of cells and RI β, from the concentrations of insulin and glucose (Matthews et al. 1985). In this method, high-scores denote low insulin sensitivity. The IR analysis is done using the following formula :

IR = insulin (μU / mL) x glucose (mmol / L) / 22.5 .

3.4.5 Evaluation of blood pressure

Blood pressure will be measured by auscultation with obedience to the American Heart Association (Perloff et al , 1993), with the subject seated in a chair for five minutes with your back supported and bare left arm at heart level . Two measurements will be made with an interval of five minutes ; the final value will be obtained by averaging these two measurements. If the first two readings differ by more than 5 mm Hg, new measures will be obtained and the avrage values will be used.

3.5 Statistical Analysis

Statistical analyzes will be performed using SAS 8.2 (Cary, USA) software. At first, we will evaluate whether there continuous variables are normally distributed; if not, a logarithmic transformation will be performed. Mixed Model will be used for comparative analysis of group averages. When interactions is significant, we will perform post-hoc analysis with Tukey's test .

3.6 Clinical Evaluation

All patients will undergo static and dynamic postural assessment involving positioning of the shoulders, asymmetries , members discrepancy, muscle shortening, scoliosis, cervical and lumbar lordosis , gait , genu varus and genu valgus , and limiting cardiovascular diseases.

3.6.1 Methodology of ergospirometry

- Evaluation of Cardiopulmonary Capacity During Maximal Progressive Exercise:

Tests will be performed at the Laboratory of Assessment and Conditioning in Rheumatology, Hospital das Clinicas, Faculty of Medicine, USP, which has all the necessary infrastructure for the operational development of the project. The site assessments will be air-conditioned room temperature, percent relative humidity and barometric pressure will be continuously monitored for conducting evaluations .
Initially an electrocardiogram will be performed at rest using the twelve standard leads (D1 , D2 , D3 , aVR , aVL , aVF , V1 , V2 , V3 , V4 , V5 , V6). Then the resting blood pressure will be measured by auscultation with a mercury column sphygmomanometer. Study participants will undergo a stress test on a treadmill (Centurion 200 , Micromed), following a ramp protocol with increased every minute in the workload (speed and / or incline) until exhaustion.
All participants will be studied in an environment with controlled temperature (20-22 °) and at least two hours after a meal .
In addition, they will be instructed not to drink caffeinated beverages and do physical activity in the 24 hours prior to the examination .
During the stress test, the cardiovascular behavior will be continuously assessed by electrocardiograph, with twelve simultaneous leads (D1 , D2 , D3 , aVR , aVL , aVF , V1 , V2 , V3 , V4 , V5 , V6). Heart rate (HR) will be recorded at rest with the child positioned on the conveyor belt at the end of each minute of the exercise test and the 1st , 2nd , 4th and 6th minute of recovery and chronotropic reserve will be calculated . Blood pressure will be always measured by the same person at rest, every two stages of exercise and the 1st , 2nd , 4th and 6th minute of the recovery period .
The assessment of maximal aerobic capacity will be made by direct measurement of the oxygen consumption at peak exercise (peak VO_2). Simultaneously to the stress test , each subject will be connected to a computerized ergospirometer (Metalyzer Model III b / breath- by- breath), through a valve system and sensor where the pulmonary ventilation (VE) will be measured every exhalation. Each respiratory cycle will be analyzed through oxygen sensors (O_2) and carbon dioxide (CO_2) the fractions of expired O_2 and

CO_2, respectively.
From the analysis of the VE and the concentrations of expired gases, oxygen consumption (VO_2) and carbon production (VCO_2) emissions will be calculated. VO_2peak will be considered as the consumption of O_2 obtained at peak exercise, when the individual could no longer maintain the speed imposed by the ergometer .
Shall be considered as maximum cardiopulmonary evaluation when each child reaches one of the following criteria (Rowland , 1993) :
a) Evidence of subjective exhaustion.
b) peak heart rate > 190 beats / min;
c) respiratory exchange ratio (RER) > 1.00 .

3.6.2 Determination of the Ventilatory Anaerobic Threshold

The ventilatory anaerobic threshold will be determined by the same evaluator, using the following criteria (Wasserman et al, 1973; Wasserman, 1984):
a) Values of ventilatory equivalent for oxygen (VE/VO_2) and lower partial pressure of oxygen at the end of expiration ($PETO_2$), ie , before beginning a gradual increase without concomitant rise in the ventilatory equivalent for carbon dioxide (VE / VCO_2);
b) loss of linearity of the relationship between oxygen consumption (VO_2) and carbon dioxide production (VCO_2) ;
c) non- linear value of the increment in respiratory exchange ratio (RER).

3.6.3 Determination of Respiratory Compensation Point

The respiratory compensation point will be determined by the same evaluator, using the following criteria (Skinner et al, 1980):
a) Lower values of ventilatory equivalent for carbon (VE/VCO_2) dioxide before beginning a gradual increase;
b) Higher partial pressure of end-tidal carbon ($PETCO_2$) dioxide before

starting to decline.

3.7 Protocol of Physical Training

3.7.2 Protocol of Physical Training Continuum

A random group of obese children will undergo aerobic exercises 2x/week, performed by the continuous method on a treadmill for 30 minutes at an intensity of 80% VO2peak. Every 3 weeks there will be an increase of 10 minutes in training, totaling a 60-minute workout in the last 3 weeks.

The choice of this protocol was based on studies that showed improvement in cardiovascular parameters in children undergoing continuous training (BLAQUET et al , 2003).

3.7.3 - Physical Training Protocol Flashing

A random group of obese children will undergo interval exercises to be performed by intermittent treadmill method as follows:

Working range - 1 minute at 100% of speed and incline of VO2peak .

Active Recovery - 3 minutes at 100% to 50% speed of VO2peak.

References

AMERICAN DIABETES ASSOCIATION (EUA). Type 2 diabetes in children and adolescents. **Pediatrics**, Chicago, v. 105, n. 3, p.671-680, Mar. 2000.

AMERICAN DIABETES ASSOCIATION POSITION STATEMENT. Diagnosis and classification of diabetes mellitus. **Diabetes Care**, Alexandria, v. 1, n. 27, suppl. 1, p.S5-S10, 01 Jan. 2004.

BAQUET, G. et al. Effects on High Intensity Intermittent Training on Peak VO2 in Prepubertal Children. **International Journal Sports Medicine**, Stuttgart, v. 23, n. 6, p.439-444, 01 Aug. 2002

BAQUET, G. et al. Endurance Training and Aerobic Fitness in Young People. **Sports Medicine,** Ronchin, France, v. 33, n.15, p.1127-1143, 2003.

BARLOW, Sarah E. et al. Expert Committee Recommendations Regarding the Prevention, Assessment, and Treatment of Child and Adolescent Overweight and Obesity: Summary Report. **Pediatrics**, Chicago, v. 120, p.S164-S192, 01 Dec. 2007

CHAN, Gilbert; CHEN, Christopher T.. Musculoskeletal effects of obesity. **Current Opinion In Pediatrics**, Colorado, v. 21, n. 1, p.65-70, 01 Feb. 2009.

CHILDHOOD obesity: an emerging public-health problem. **The Lancet**, London, v. 9272, n. 357, p.1989-1989, 23 June 2001.

COMMITTEE on nutrition: Prevention of pediatric overweight and obesity. **Pediatrics**, Chicago, v. 112, p.424-430, 2003.

COOK, Stephen et al. Prevalence of a metabolic syndrome phenotype in adolescents: findings from the Third National Health and Nutrition Examination Survey, 1988-1994. **Arch Pediatr Adolesc Med**, Chicago, v. 157, n. 8, p.821-827, Aug. 2003.

DANIELS, Stephen R. et al. Overweight in Children and Adolescents: Pathophysiology, Consequences, Prevention, and Treatment. **Circulation**, Dallas, v. 111, p.1999-2012, 19 Apr. 2005.

EXECUTIVE Summary of the Third Report of the National Cholesterol Education Program (NCEP) Expert Panel on Detection, Evaluation, and Treatment of High Blood Cholesterol in Adults (Adult Treatment Panel III **Jama**, Chicago, v. 285, n. 19, p.2486-2497, 16 May 2001.

FOSS, Merle L.; KETEVIAN, Steven J.. **Fox:** Bases Fisiológicas do Exercício e do Esporte. 6. ed. Rio de Janeiro: Guanabara Koogan, 2000.

GIBALA, M. J.; MCGEE, S. L.. Metabolic Adaptations to Short-term High-Intensity Interval Training: A Little Pain for a Lot of Gain? **Exercise Sports Science. Rev.**, Massachussets, v. 36, n. 2, p.58-63, 25 Apr. 2008.

GORAN, M. I.; GOWER, B. A.. Longitudinal study on pubertal insulin resistance. **Diabetes**, New York, v. 11, n. 50, p.2444-2450, Nov. 2001.

GORMALLY, Jim et al. The assessment of binge eating severity among obese persons. **Addictive Behaviors,** v. 7, p. 47-55, 1982.

INSTITUTO BRASILEIRO DE GEOGRAFIA E ESTATÍSTICA. **Pesquisa de orçamentos familiares 2002-2003**: antropometria e análise do estado nutricional de crianças e adolescentes no Brasil. Rio de Janeiro: IBGE, 2006. 140 p

LOURENÇO, B. H. et al. Binge eating symptoms, diet composition and metabolic characteristics of obese children and adolescents. **Appetite**, London, v. 50, p.223-230, May 2008. Trimestral.

OGDEN, Cynthia L. et al. Prevalence of overweight and obesity in the United States, 1999–2004. **Jama**, Chicago, v. 295, n. 13, p.1549-1555, 5 Apr. 2006.

PERLOFF, D. et al. Human blood pressure determination by sphygmomanometry. **Circulation,** Dallas, v. 88, n.5, p.2460-70, Nov. 1993.

PINTO, AL SÁ et al. Musculoskeletal findings in obese children. **Journal of Paediatrics and Child Health,** Austrália v. 42, p. 341-344, Jan. 2006.

REAVEN, G. M. et al. Plasma insulin, C-peptide, and proinsulin concentrations in obese and nonobese individuals with varying degrees of glucose tolerance. **J clin Endocrinol Metab**, Charlotte, Usa, v. 76, n. 1, p.44-48, Jan. 1993.
